# Supplementary material for: Automated Large-Scale Production of Paclitaxel Loaded Mesenchymal Stromal Cells for Cell Therapy Applications
Source: Pharmaceutics. 2020 Apr 30;12(5):411. doi: 10.3390/pharmaceutics12050411 (PMC7284468; doi:10.3390/pharmaceutics12050411)
Supplement: Supplementary file 1 [file pharmaceutics-12-00411-s001.pdf]

pharmaceutics

MDPI

Supplementary Materials: Automated Large-Scale Production of Paclitaxel Loaded Mesenchymal Stromal Cells for Cell Therapy Applications

Daniela Lisini, Sara Nava, Simona Frigerio, Simona Pogliani, Guido Maronati, Angela Marcianti, Valentina Coccè, Gianpietro Bondiolotti, Loredana Cavicchini, Francesca Paino, Francesco Petrella, Giulio Alessandri, Eugenio A. Parati and Augusto Pessina

Table S1. MSCs-PTX viability over time. Viability of both fresh and freeze/thawed MSCs-PTX obtained with bioreactor, assessed after 7, 14 and 21 days from re-seeding in plastic flasks. In this period of time medium was changed every 3 days; the cells have never been detached, due to the lost of their duplication capacity and the failure to reach confluence. Results were expressed as mean ± SD of *n* = 3 experiments. F: fresh, FT: freeze/thawed.

	**Viability MSCs-PTX (F) (mean ± SD)**	**Viability MSCs-PTX (FT) (mean±SD)**
7 days	95.3% ± 3.1%	92.3% ± 2.1%
14 days	93.1% ± 1.9%	91.6%% ± 3.3%
21 days	93.7% ± 3.5%	89.7% ± 2.9%

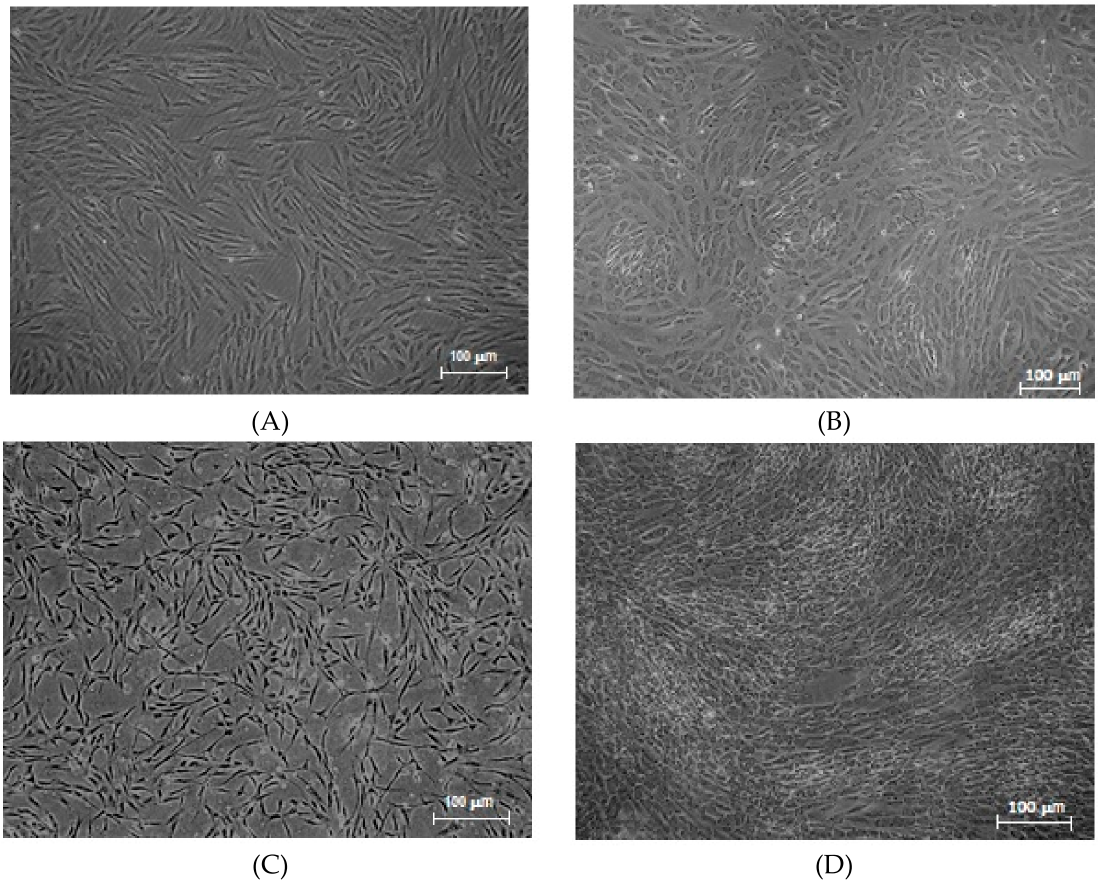

Figure S1. MSCs and MSCs-PTX morfology. (**A**) MSCs, cultured in plastic flasks (pre-treated with fibronectin; passage 3); (**B**) MSCs-PTX cultured in plastic flasks (pre-treated with fibronectin; cells were loaded with PTX at the end of passage 3 and the image was taken 24 h after); (**C**) MSCs expanded in bioreactor; (**D**) MSCs expanded and loaded with PTX (for 24 h) in bioreactor. Magnification 5×.

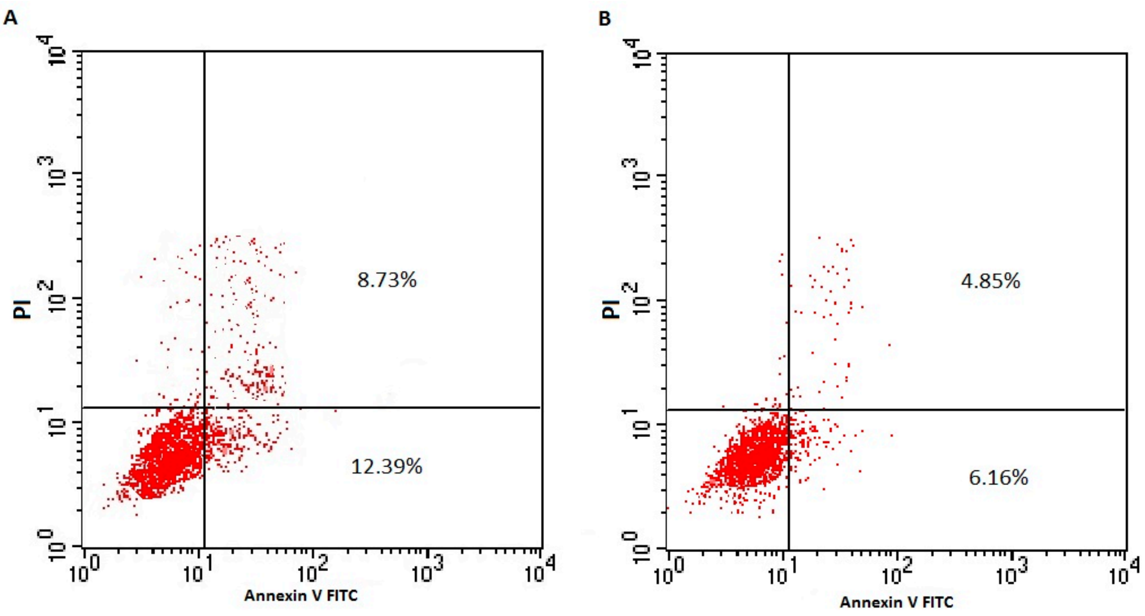

Figure S2. Flow cytometry analysis of apoptosis and necrosis. The dot plot diagrams represent typical apoptotic and necrotic cell populations detected by Annexin V-FITC and PI staining. **A**) MSCs-PTX; **B**) MSCs. The lower left quadrants of the panels show viable intact cells (double negative); the upper right quadrants show necrotic cells (double positive); the lower right quadrants represents apoptotic cells, positive for Annexin V and negative for PI. Diagrams are representative for one of 6 repeated experiments.

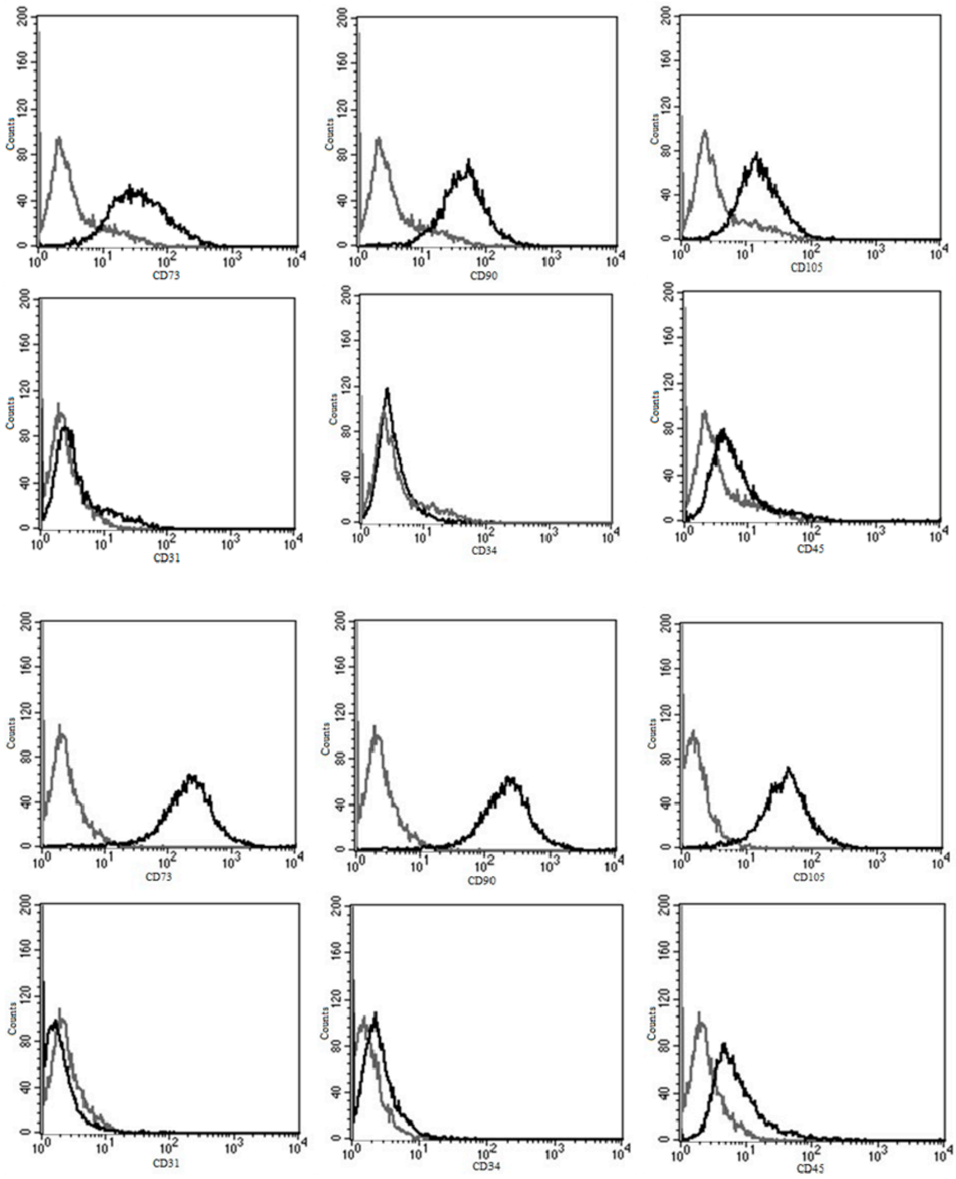

A

B

Figure S3. Flow cytometry analysis of a representative MSCs line, before and after expansion and loading with PTX in the bioreactor. (**A**) analysis of the typical positive MSC markers CD73 (upper line,left), CD90 (upper line, center) and CD105 (upper line, right) and the negative MSCs markers CD31 (lower line, left), CD34 (lower line, center) and CD45 (lower line, right); (**B**) analysis of the correspondent MSCs-PTX for CD73 (upper line, left), CD90 (upper line, center) and CD105 (upper line, right), CD31(lower line, left), CD34 (lower line, center) and CD45 (lower line, right).
